# Supplementary material for: Oxygen-catalysed sequential singlet fission
Source: Nat Commun. 2019 Nov 15;10:5202. doi: 10.1038/s41467-019-13202-5 (PMC6858316; doi:10.1038/s41467-019-13202-5)
Supplement: Supplementary file 1 — Supplementary Information [file 41467_2019_13202_MOESM1_ESM.pdf]

SUPPLEMENTARY INFORMATION

Oxygen-Catalysed Sequential Singlet Fission

Wollscheid et al.

Supplementary Note 1: Time-Dependent Concentrations

The definitions $k_1' \equiv k_1[\,^3O_2]$, $k_2' \equiv k_2[S_0]$, $k_{SF}' \equiv k_{SF}[S_0]$ and $k_{tot} \equiv k_R + k_1' + k_{SF}'$ are applied for shortness. The reaction schemes are shown in Figure 2, panels a and b. The rate equations can be integrated analytically, resulting in the following time-dependent concentrations:

$$[\boldsymbol{S_1}](\boldsymbol{t}) = [\boldsymbol{S_1}]_{\boldsymbol{0}}\boldsymbol{e}^{-\boldsymbol{k_{tot}t}} \tag{1}$$

$$[\,^{1}\boldsymbol{O}_2](\boldsymbol{t}) = \frac{\boldsymbol{k_1'}[\boldsymbol{S_1}]_{\boldsymbol{0}}}{\boldsymbol{k_{tot}} - \boldsymbol{k_2'}}\left\{\boldsymbol{e}^{-\boldsymbol{k_2't}} - \boldsymbol{e}^{-\boldsymbol{k_{tot}t}}\right\} \tag{2}$$

$$\begin{aligned}[\boldsymbol{T_1}]_{\boldsymbol{total}}(\boldsymbol{t}) = &\left\{-\frac{(\boldsymbol{k_1'} + 2\boldsymbol{k_{SF}'})[\boldsymbol{S_1}]_{\boldsymbol{0}}}{\boldsymbol{k_{tot}} - \boldsymbol{k_T}} + \frac{\boldsymbol{k_1'k_2'}[\boldsymbol{S_1}]_{\boldsymbol{0}}}{(\boldsymbol{k_{tot}} - \boldsymbol{k_2'})(\boldsymbol{k_{tot}} - \boldsymbol{k_T})}\right\}\boldsymbol{e}^{-\boldsymbol{k_{tot}t}} \\ &- \frac{\boldsymbol{k_1'k_2'}[\boldsymbol{S_1}]_{\boldsymbol{0}}}{(\boldsymbol{k_{tot}} - \boldsymbol{k_2'})(\boldsymbol{k_2'} - \boldsymbol{k_T})}\boldsymbol{e}^{-\boldsymbol{k_2't}} \\ &+ \left\{\frac{(\boldsymbol{k_1'} + 2\boldsymbol{k_{SF}'})[\boldsymbol{S_1}]_{\boldsymbol{0}}}{\boldsymbol{k_{tot}} - \boldsymbol{k_T}} + \frac{\boldsymbol{k_1'k_2'}[\boldsymbol{S_1}]_{\boldsymbol{0}}}{(\boldsymbol{k_2'} - \boldsymbol{k_T})(\boldsymbol{k_{tot}} - \boldsymbol{k_T})}\right\}\boldsymbol{e}^{-\boldsymbol{k_Tt}}\end{aligned} \tag{3}$$

Timescales

The reaction rates *k* for homogeneous SF and energy transfer to $^3O_2$ depend on the concentration of the reactants, which are TIPS-Pn and $^3O_2$, respectively. This is shown in the main text (Equation 4). Thus, the timescales can be calculated as $\tau = 1/k$ for any given concentration. For homogeneous SF, the examined concentration range of TIPS-Pn is 0.02 to 160 mM. Using the reported literature value of $k_{SF} = 2.18 \times 10^9$ (M s)$^{-1}$ (Supplementary Table 3), this corresponds to a timescale of 2.30 µs to 2.87 ns. Regarding the energy transfer step, the reported literature rate of $k_1 = 3.12 \times 10^{10}$ (Ms)$^{-1}$ for atmospheric conditions ([$^3O_2$] = 1.81 mM) corresponds to a time constant of τ_1 = 17.7 ns. In deaerated solutions, energy transfer does not occur at all.

Diffusion length

The diffusion length L can be calculated as $\mathrm{L} = \sqrt{D\tau}$ [1] with the diffusivity D and the lifetime τ. D can be approximated using the Stokes-Einstein-Relation $\mathrm{D} = \frac{k_B T}{6\pi\eta r}$ with r(O_2) = 2 Å, r(TIPS-Pn) = 4.13 Å and η = 0.48 x 10^{-3} kg/(ms). [2-4] The lifetimes τ were taken from literature[5] in the case of $^1O_2$ or calculated as $1/k_R$ (Supplementary Table 3), respectively. Thus, a Diffusion length of 246 nm for oxygen and 3.6 nm for TIPS-Pn is obtained. This shows that the diffusion length increases by a factor of 68.3 for oxygen compared to TIPS-Pn.

Supplementary Note 2: Stationary Absorption Spectra

UV-Vis absorption spectra were obtained using a Shimadzu UV-2600 spectrophotometer using a 10 µm and 1 mm fused-silica cell (Starna) for $c \geq 1$ mM and $c < 1$ mM, respectively. TIPS-Pn shows a strong vibronic structure characteristic for acenes (Supplementary Figure 1a). The decrease of the relative intensity at 640 nm is explained by the absorption exceeding the dynamic range of the spectrometer. For the second vibronic peak at 590 nm, a molar extincion coefficient of (2.22 ± 0.03) 10^4 l (mol cm)$^{-1}$ has been determined (Supplementary Figure 1c). $TDCl_4$ shows two bands centered at about 650 and 420 nm. The first absorption band shows prominent structure with vibronic transitions at 680, 620 and ≈580 nm (Supplementary Figure 1b)). The second absorption feature has an almost structureless bandshape. It reaches maximum intensity at 420 nm and exhibits a small shoulder at 390 nm. For both compounds, the absorption spectrum was measured for concentrations ranging from 0.1 to 100 mM. No significant spectral changes or additional features were observed, strongly suggesting that neither aggregation nor any other kind of self-interaction take place.

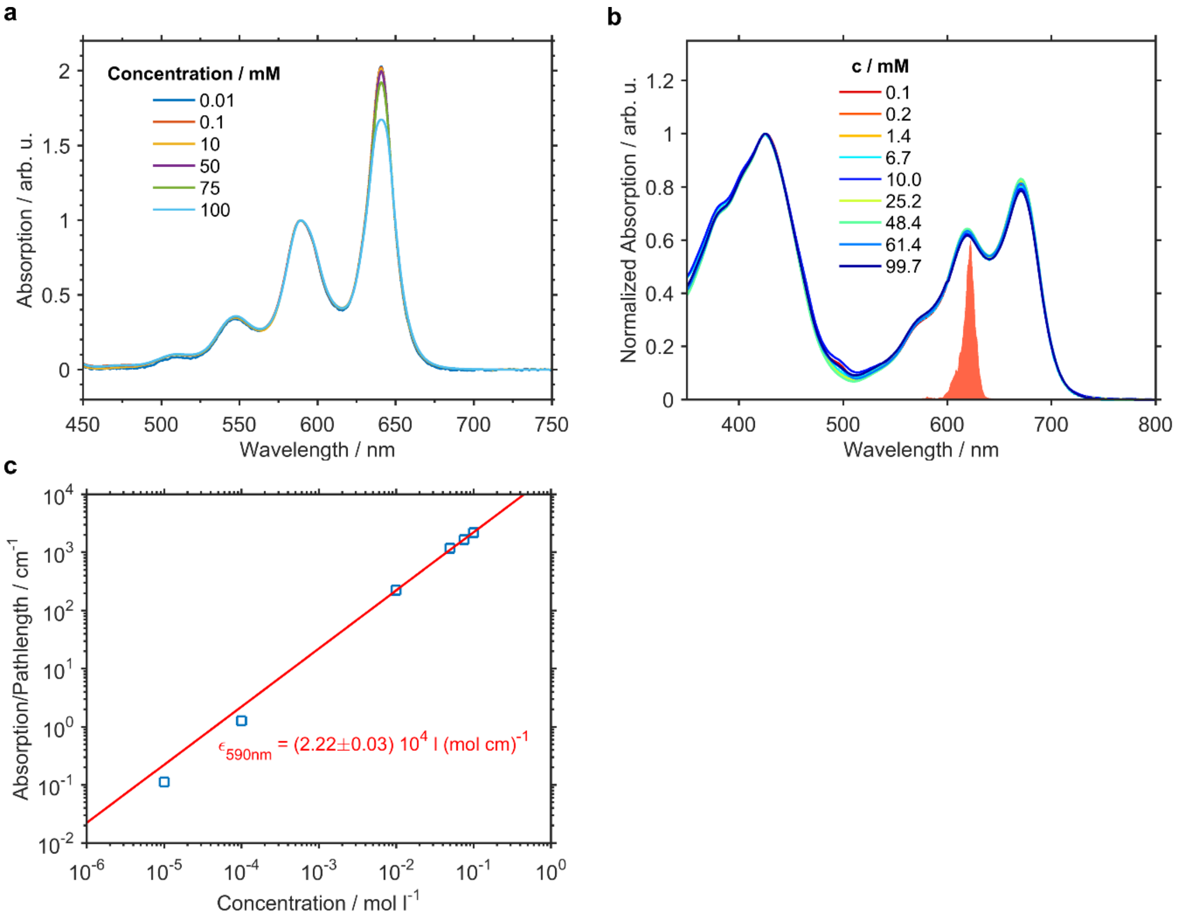

Supplementary Figure 1: *Absorption Spectra of a) TIPS-Pn and b) $TDCl_4$ at selected concentrations. No change in the spectral shape is observable, which speaks for no self-interactions taking place even at high concentrations. c) The concentration dependent*

absorption of TIPS-Pn at 590 nm shows a linear dependence, further supporting the lack of self-interaction. A molar extinction coefficient of (2.22 ± 0.03) 10^4 l mol^{-1} cm^{-1} is determined.

Photostability

The stationary spectra of TIPS-Pn and TDCl4 before and after transient absorption measurements show a perfect overlap, thus displaying great photostability (Supplementary Figure 2a and b). In contrast to this, unsubstituted pentacene degrades almost completely when excited for 2h under measurement conditions (Supplementary Figure 2c). Measurements were carried out with a mean energy per pulse of about 400 nJ at a 2 kHz repetition rate. The spot diameter was 0.3 mm. TIPS-Pn, $TDCl_4$ and pentacene were excited at 680, 620 and 575 nm, respectively.

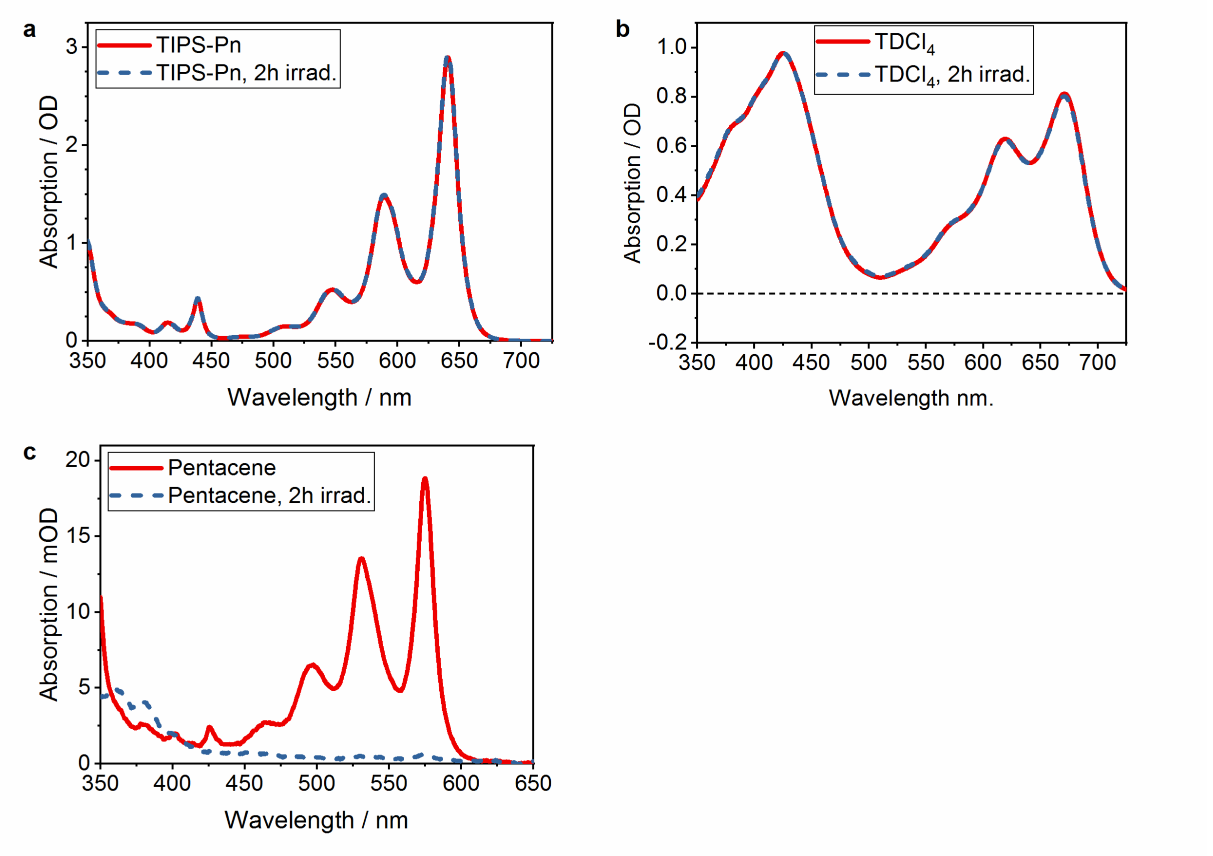

Supplementary Figure 2: *Absorption Spectra of a) TIPS-Pentacene in THF, b) $TDCl_4$ in toluene and c) Pentacene in THF before and after irradiation for 2h under ambient measurement conditions.*

Supplementary Note 3: Stationary Fluorescence Spectra

The stationary fluorescence spectrum of a dilute solution of TIPS-Pn shows two peaks at 650 and 710 nm and mirrors the vibronic structure of the absorption spectrum (Supplementary Figure 3a). For increasing concentrations, the intensity of the peak at 650 nm decreases and a slight redshift is observed. This behaviour can be explained by the small Stokes-shift observed for TIPS-Pn, which results in a self-absorption of the emission, modelled quantitatively in (Supplementary Figure 3c). $TDCl_4$ shows a fluorescence maximum at 730 nm in dilute solutions. In contrast to steady-state absorption, no obvious vibronic structure can be recognized in the fluorescence spectrum (Supplementary Figure 3b).

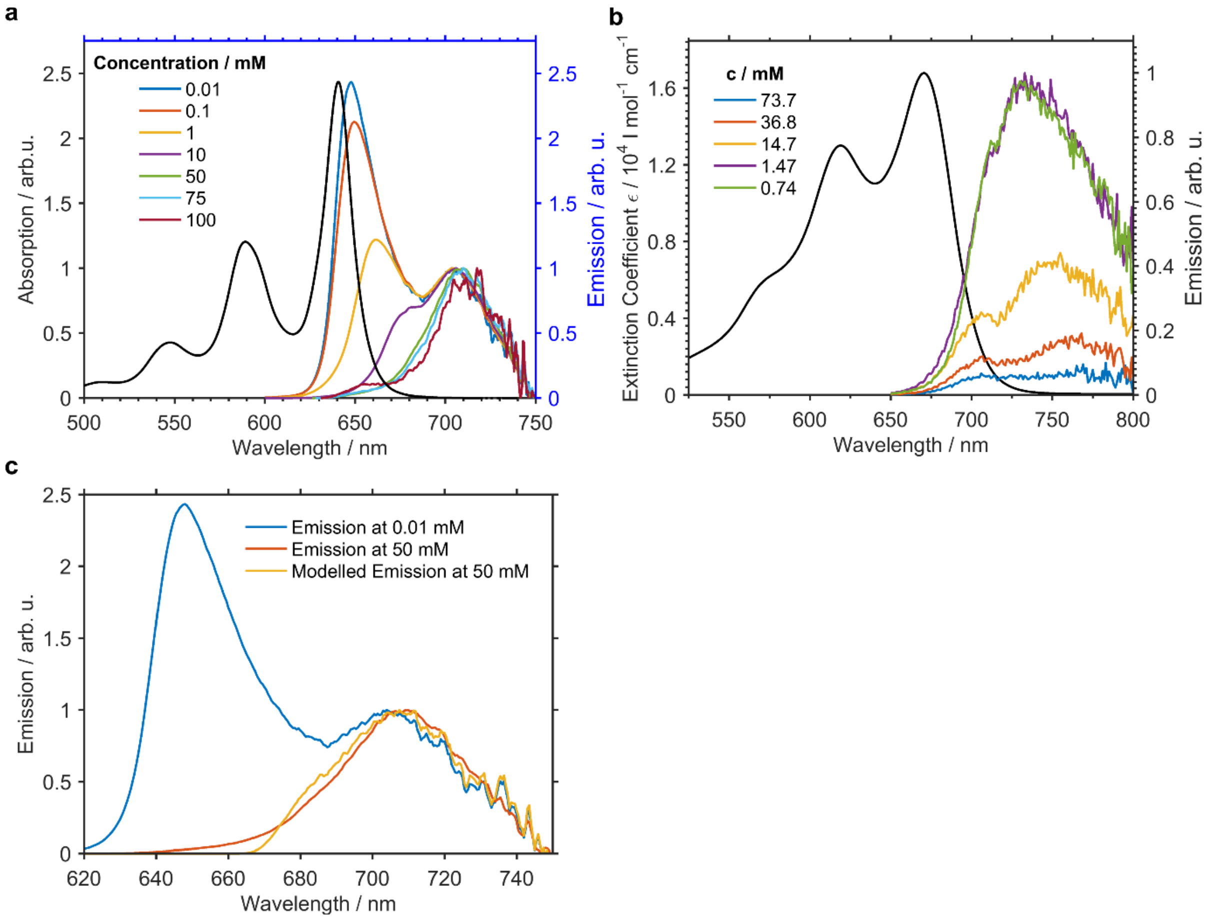

Supplementary Figure 3: *Stationary fluorescence spectra at selected concentrations for a) TIPS-Pn, normalized at the peak centred at 700 nm, and b) $TDCl_4$. c) Modelling of the inner filter effect for TIPS-Pn by multiplication of the transmission (obtained for an assumed path of 20 µm and c=50 mM) with the emission spectrum obtained for 0.01 mM.*

The emission spectra of $TDCl_4$ demonstrate concentration-dependent fluorescence quenching, overcompensating the expected increase in the "front-face" fluorescence with increasing chromophore concentration. The fluorescence quenching is accompanied by small spectral changes, namely a seemingly redshift of the maximum and the appearance of a local minimum at 720 nm at higher concentrations. This wavelength coincides with a strong ESA (Supplementary Figure 6 d-f) and

is thus attributed to a re-absorption of the spontaneously emitted photons. Thus, spectral changes observed in the stationary fluorescence of TIPS-Pn and $TDCl_4$ can be explained by inner filter effects and no assumptions of additional states have to be made, which is consistent with the TA measurements.

Supplementary Note 4: Time-Resolved Fluorescence

Time-resolved fluorescence obtained by time-correlated single photon counting shows a monoexponential behaviour for both TIPS-Pn and $TDCl_4$ (Supplementary Figure 4a and b). The respective fluorescence decay rates increase proportional to concentration, in good agreement with the total singlet decay rate obtained by TA (Supplementary Figure 4c and d). As no additional component is observed, it can be concluded that the initial singlet is the only observable emissive species.

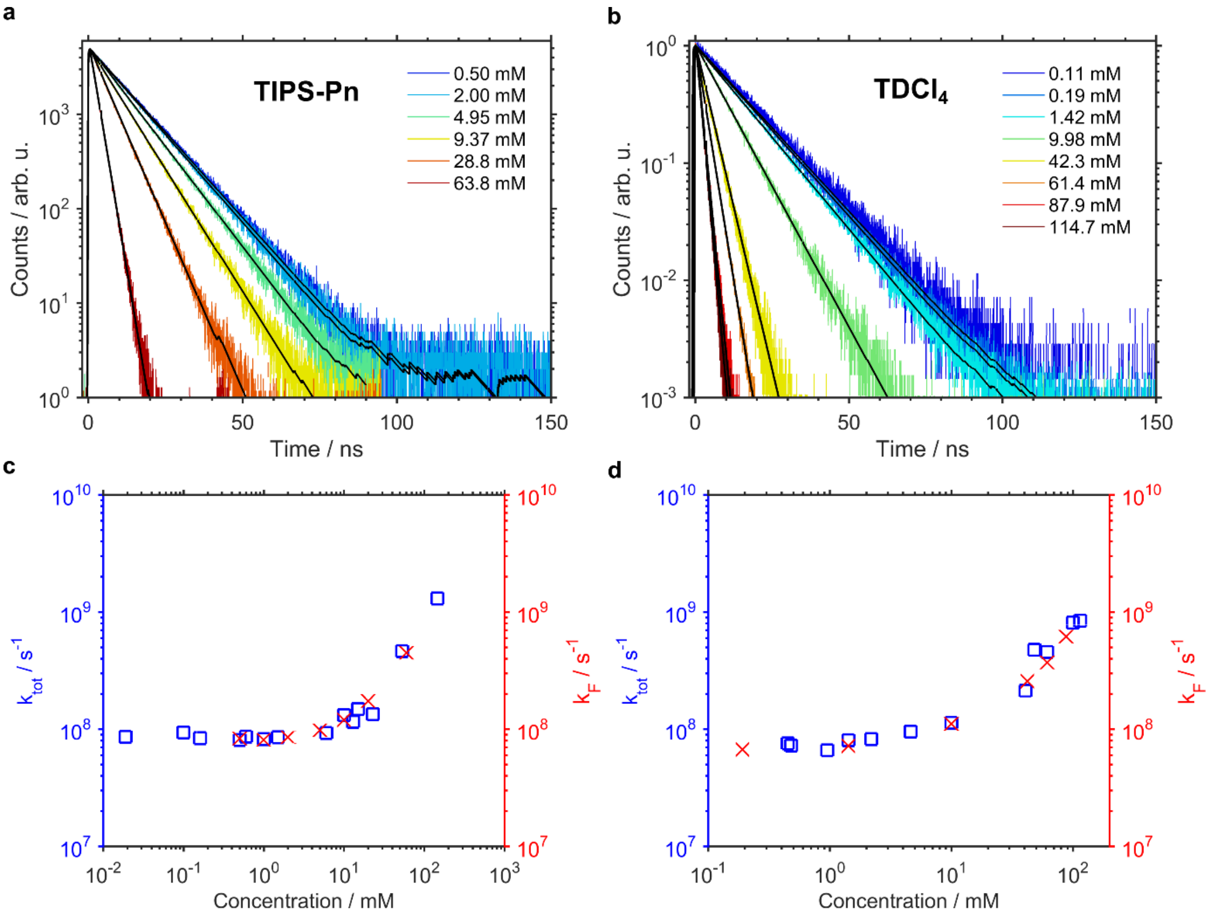

Supplementary Figure 4: *Top row: Concentration dependent fluorescence decay for a) TIPS-Pn and b)* $TDCl_4$ *with respective fits. Bottom row: Comparison of total singlet decay rates* k_{tot} (blue squares) *obtained by TA and fluorescence decay rates* k_F (red squares) *for c) TIPS-Pn and d)* $TDCl_4$*.*

Supplementary Note 5: Additional Transient Absorption Results

Oxygen Dependent Spectral Evolution in TIPS-Pn

In Supplementary Figure 5, the transient spectra of 0.5 mM TIPS-Pn in THF are compared under ambient (panel a) and oxygen-free conditions (panel b). While the triplet signal is clearly visible at late delays in the former case, it is negligibly small in the oxygen-free solution. This indicates that the triplet is formed almost exclusively by oxygen-assisted sequential SF. Furthermore, the triplet decay is accelerated from 28.2 µs (oxygen-free) to 1.88 µs (ambient conditions, compare also Figure 3).

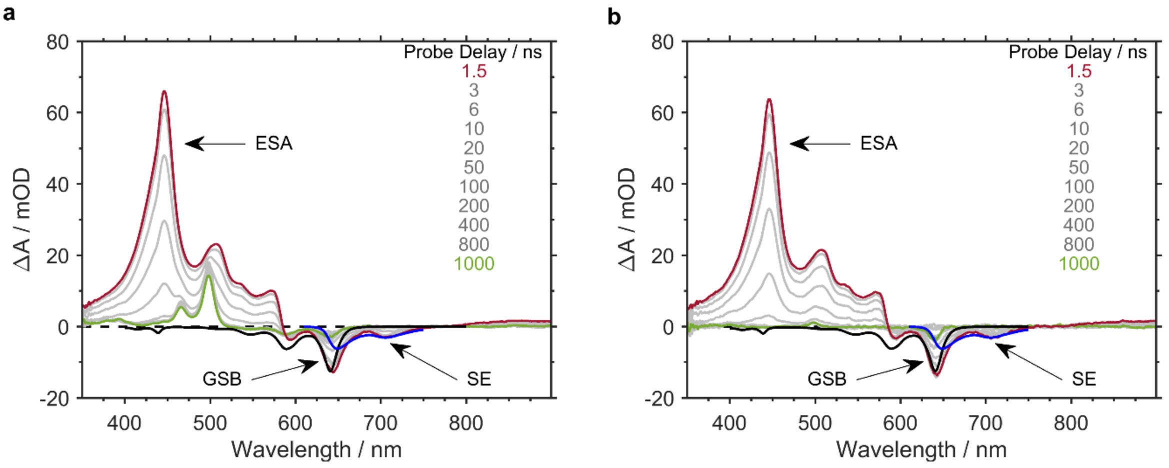

Supplementary Figure 5: *Transient spectra of 0.5 mM TIPS-Pn at selected probe delays in a) THF stored under ambient conditions and b) deaerated THF.*

In order to further confirm the role of oxygen in triplet formation, the solvent was enriched with oxygen by bubbling O_2 through a 1.5 mM solution of TIPS-Pn in THF for 10 min (Supplementary Figure 6). In this case, it is clearly seen that the highest intensity of the triplet signal is reached at earlier probe delays (95 ns versus 144 ns, respectively) and is more intense by a factor of 1.59. Furthermore, the singlet lifetime $k_{tot} = 1/\tau_1$ decreases from 11.5 ns (ambient) to 8.3 ns (O_2-enriched). This is directly linked to the increase of the $^3O_2$ concentration, as k_{SF} and k_R must remain constant. Thus, the difference between k_{tot} for O_2-enriched and ambient conditions yields (see also Equation 4 in the main text)

$$\Delta k_{tot} = k_1\left(\left[\,^3O_2\right](\text{enriched}) - \left[\,^3O_2\right](\text{ambient})\right) \tag{4}$$

Using the known values for k_1 as well as the known oxygen concentration for atmospheric conditions (Supplementary Table 3), the concentration for the O_2-enriched sample can be calculated as 5.0 mM. Subsequently, the quantum yield for the energy transfer process can be calculated as

$$QY = \frac{k_1\left[\,^3O_2\right]}{k_{tot}} \tag{5}$$

Based on that, the quantum yield doubles from 21.6 % to 43.4 % when increasing the $^3O_2$ concentration from 1.81 to 5.0 mM. Furthermore, the time constant τ_2, which is related to heterogeneous SF, remains virtually constant with 51 ns (ambient) and 49 ns (O2-enriched), respectively. This is to be expected, as the sequential mechanism implies a dependence solely on chromophore concentration (compare with Figure 2). The triplet lifetime decreases from 1.35 µs (ambient) to 520 ns (O_2-enriched). Together with the results of the oxygen-free measurements, it can be concluded that O_2 impacts not only triplet formation, but its relaxation as well.

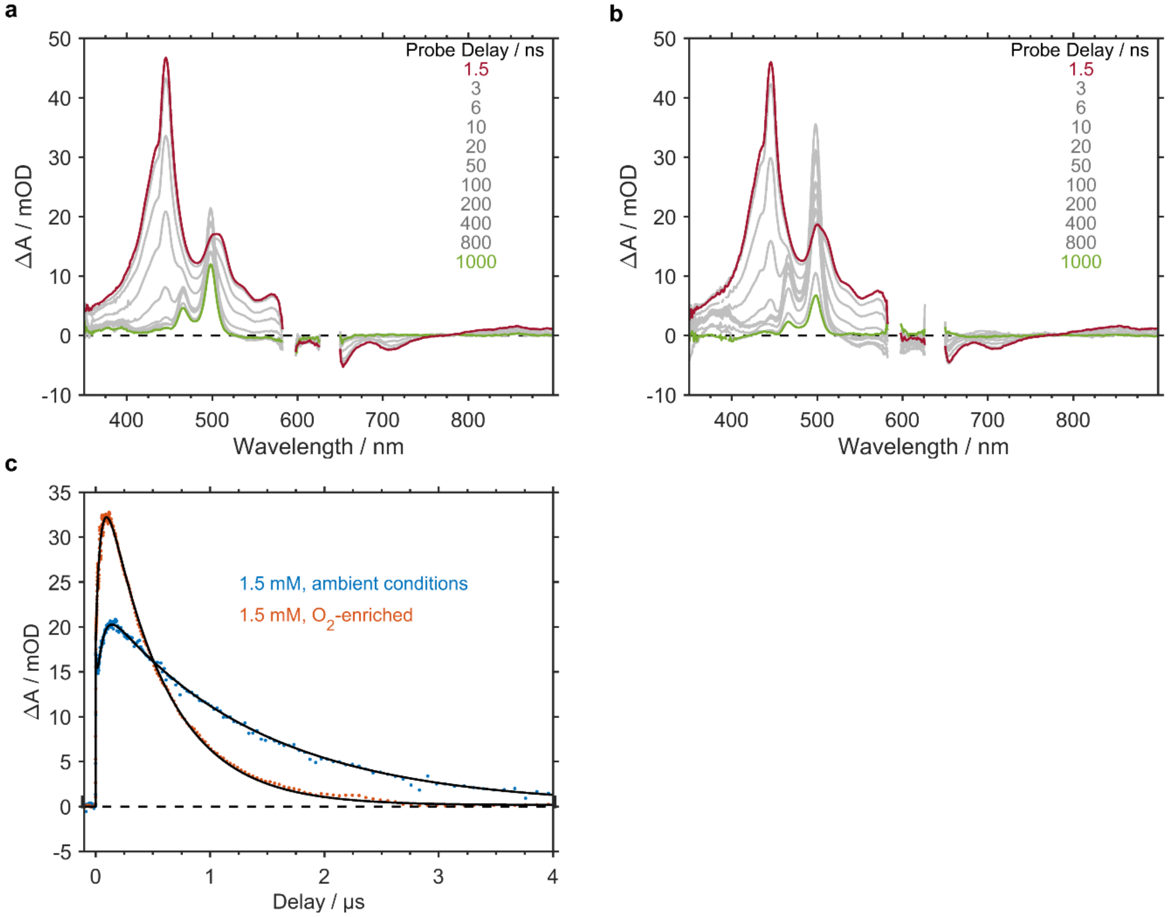

Supplementary Figure 6: *Transient spectra of 1.5 mM TIPS-Pn at selected probe delays in a) THF stored under ambient conditions and b) Oxygen-enriched THF. The latter was prepared by passing O_2 through the solution for 10 min. c) Transients at 500 nm of 1.5 mM TIPS-Pn in THF under ambient and oxygen-enriched conditions with corresponding fits.*

Effects of oxygen on the singlet decay of TIPS-tetracene

The triplet energy of TIPS-tetracene (Figure 6) does not allow for sequential SF. This is confirmed by TA measurements of 0.8 mM TIPS-tetracene in deaerated and oxygen-equilibrated THF. In both cases, an initially formed singlet ESA with peaks at 415 and 470 nm is observed (Supplementary Figure 7a and b). Within 40 ns, a triplet ESA centred at 510 nm is formed. Under ambient conditions, this signal decays completely whereas it remains constant in a deaerated solution. A global fit shows a slight acceleration of a monoexponential singlet decay from 13.3 ± 0.1 ns (deaerated) to 10.8 ±0.1 ns (ambient), as seen for the transients at 420 nm (Supplementary Figure 7c). This corresponds to an acceleration of the reaction rate of 1.72 10^7 s^{-1}. The triplet signal decays with a time constant of 169 ± 6 ns under ambient conditions. In a deaerated solution, no decay can be observed within 4 µs (Supplementary Figure 7d), comparable to TIPS-Pn (Figure 3b).

The acceleration of the singlet decay for ambient conditions is in accordance with the diffusion rate of molecular oxygen (Supplementary Table 3). This suggests that the same energy transfer process observed in TIPS-Pn and $TDCl_4$ takes place (Supplementary Tables 3 and 4). However, in contrast to the results presented in the main text (Figure 3b), TIPS-tetracene shows no rising component in the triplet signal intensity. Thus, it can be concluded that no heterogeneous SF occurs. Both findings are in agreement with the presented model for sequential SF as well as calculated energy levels (Figure 6).

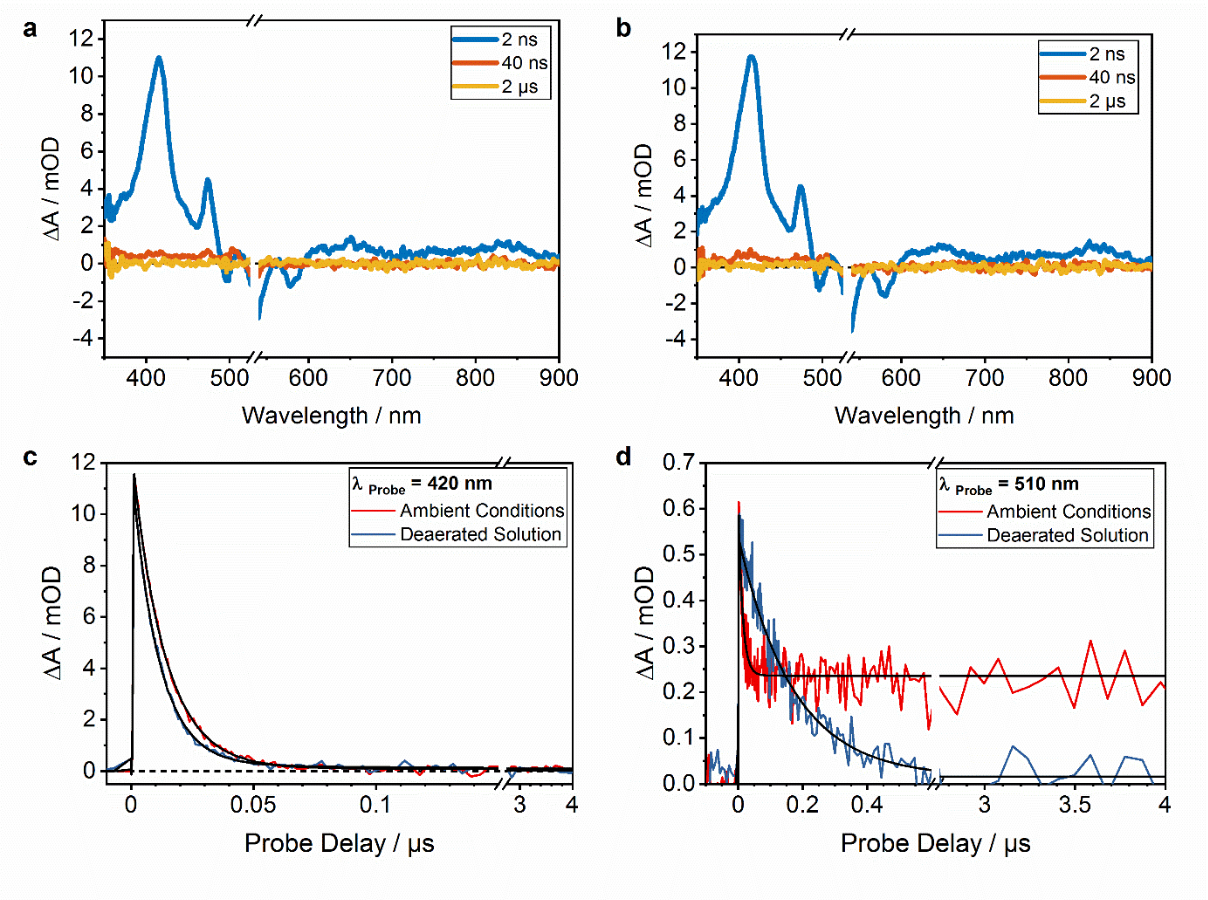

Supplementary Figure 7: *Transient spectra at selected delays for TIPS-tetracene in THF a) under ambient conditions and b) in a deaerated solvent. c) Transients at 420 nm for 0.8 mM TIPS-tetracene in THF under ambient conditions (red) and in a deaerated solvent (blue) with respective fits. d) Transients at 510 nm for 0.8 mM TIPS-tetracene in THF under ambient conditions (red) and in a deaerated solvent (blue) with respective fits.*

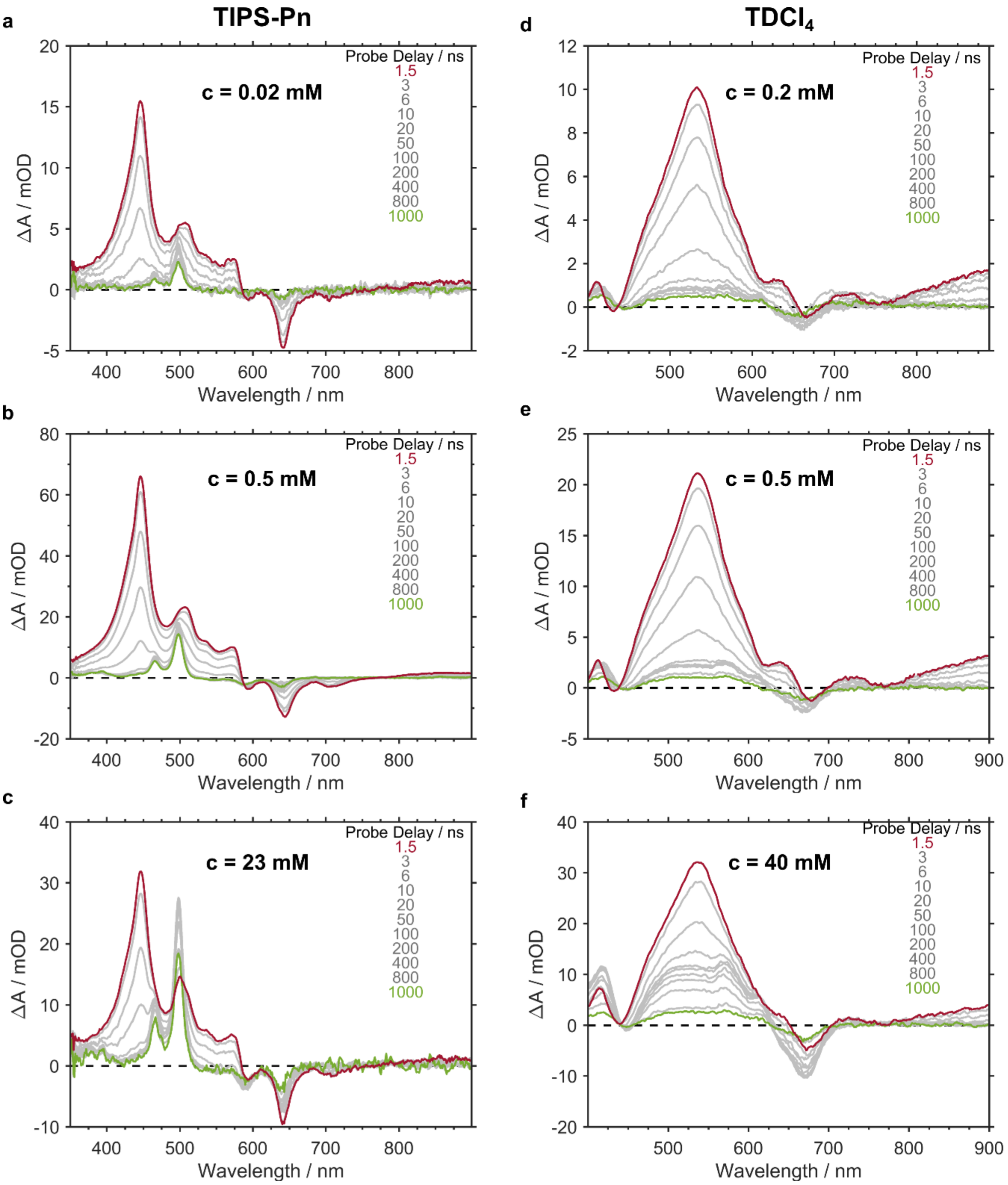

Supplementary Figure 8: *Transient spectra at selected probe delays for selected concentrations of a)-c) TIPS-Pn and d)-f)* $TDCl_4$ *in ambient conditions.*

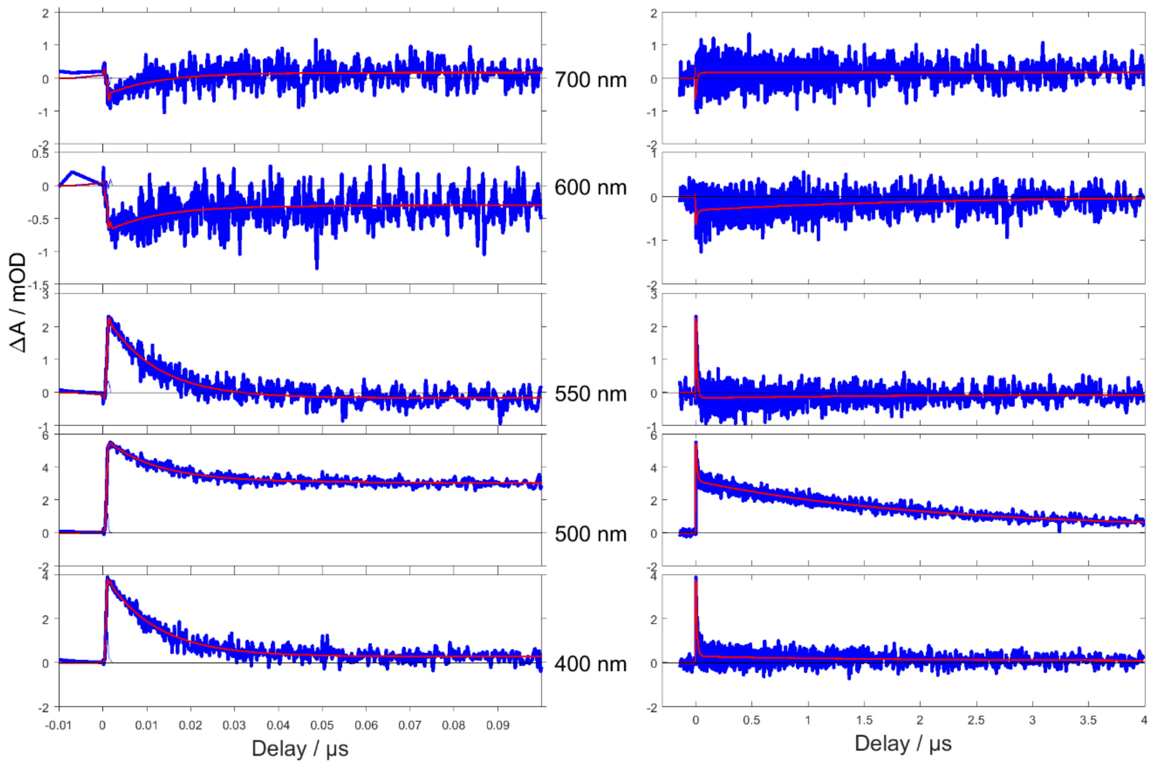

Supplementary Figure 9: *Selected kinetic traces (blue) with respective biexponential fits (red) for 0.02 mM TIPS-Pn in THF shown for initial 100 ns (left) and full timescale (4 µs).*

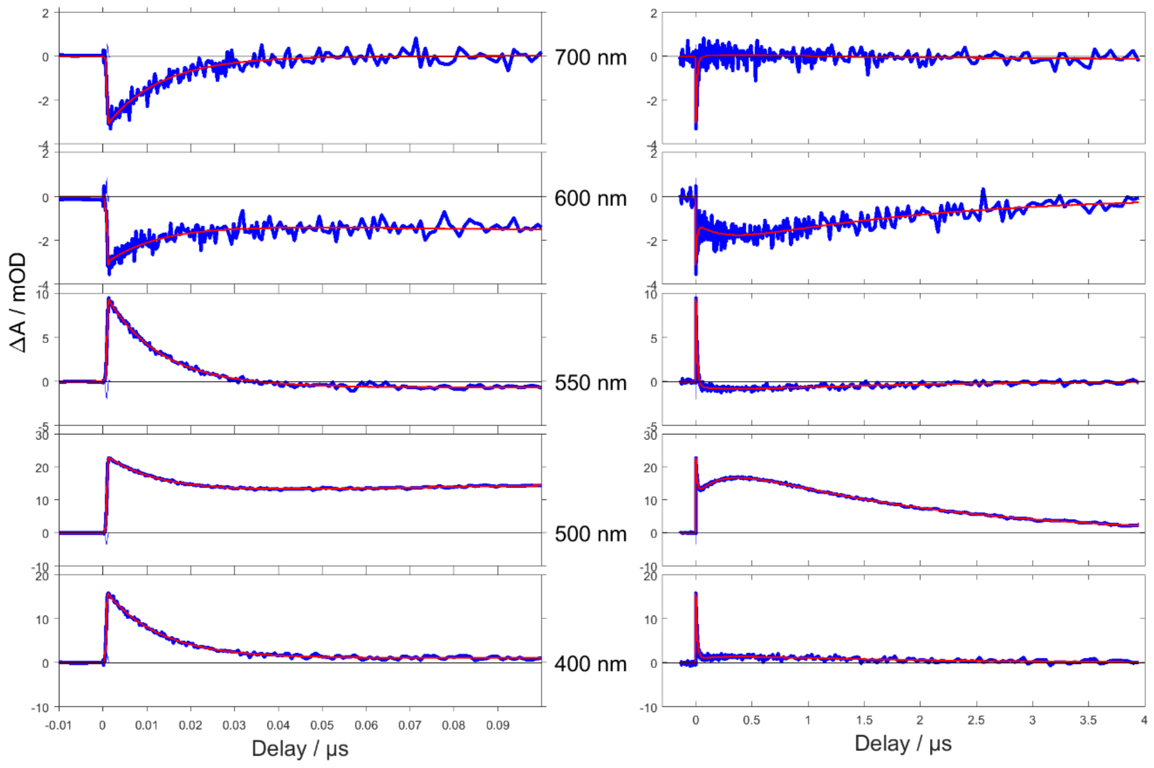

Supplementary Figure 10: *Selected kinetic traces (blue) with respective triexponential fits (red) for 0.5 mM TIPS-Pn in THF shown for initial 100 ns (left) and full timescale (4 µs).*

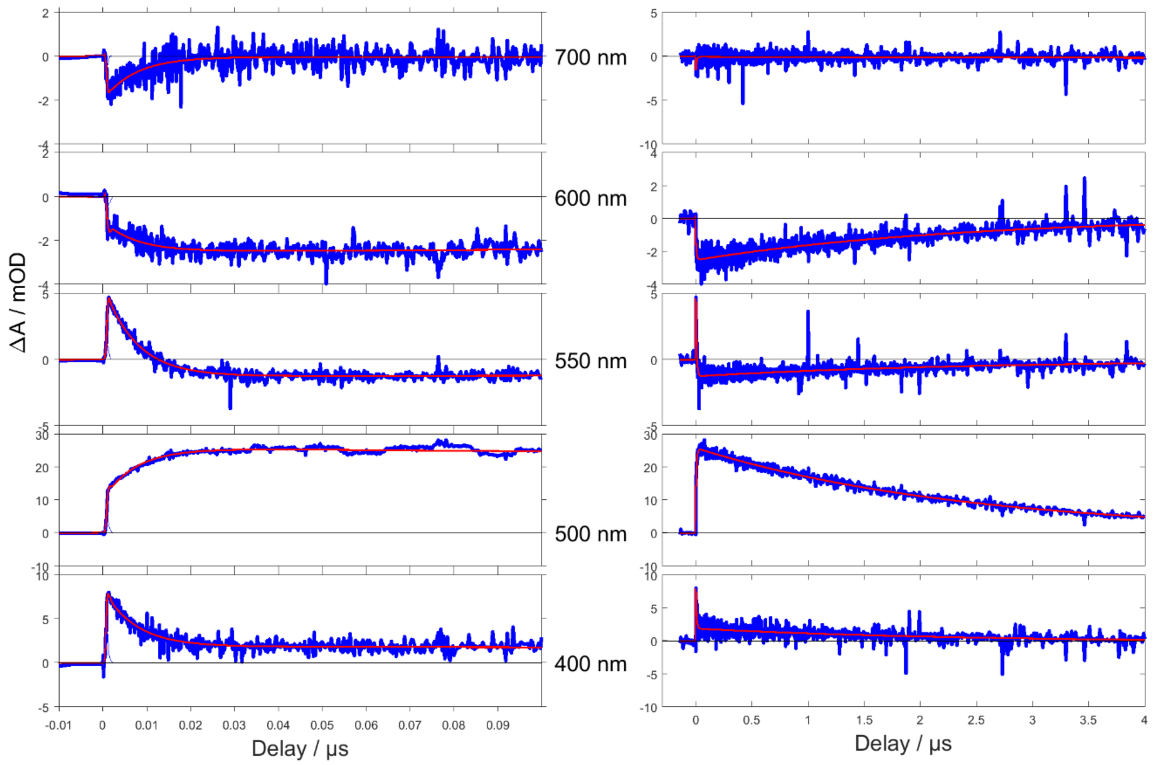

Supplementary Figure 11: *Selected kinetic traces (blue) with respective biexponential fits (red) for 23 mM TIPS-Pn in THF shown for initial 100 ns (left) and full timescale (4 μs).*

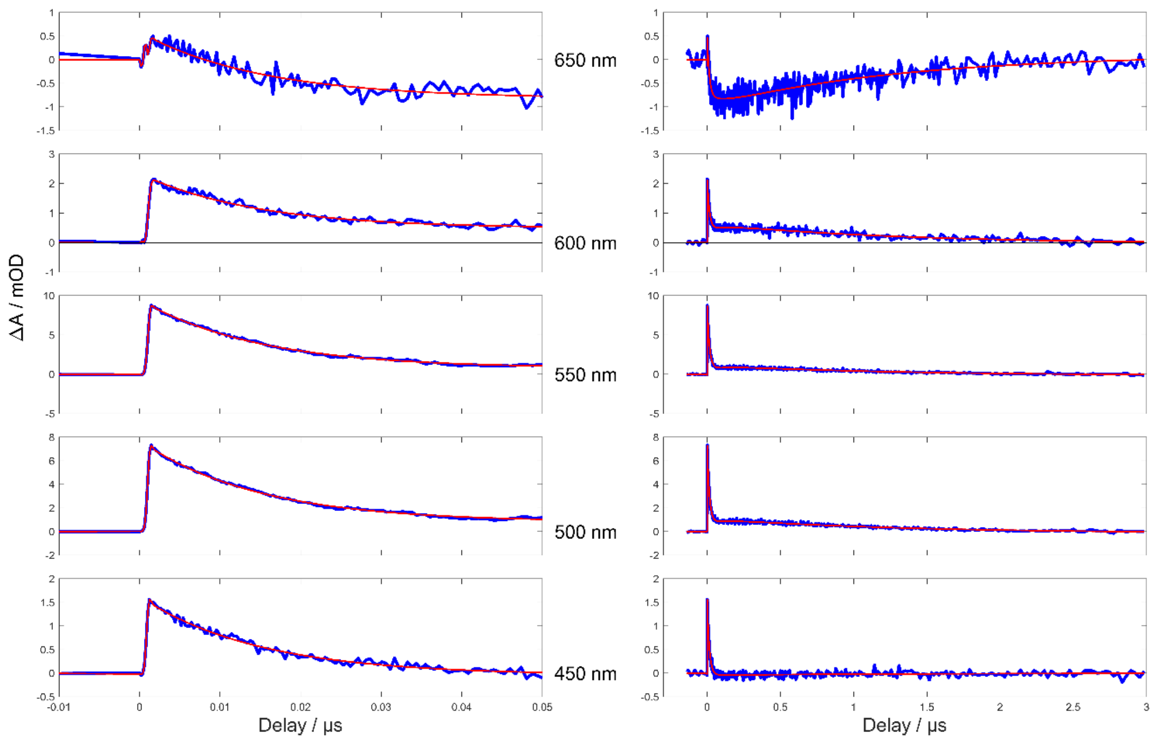

Supplementary Figure 12: *Selected kinetic traces (blue) with respective triexponential fits (red) for 0.2 mM $TDCl_4$ in toluene shown for initial 100 ns (left) and full timescale (3 µs).*

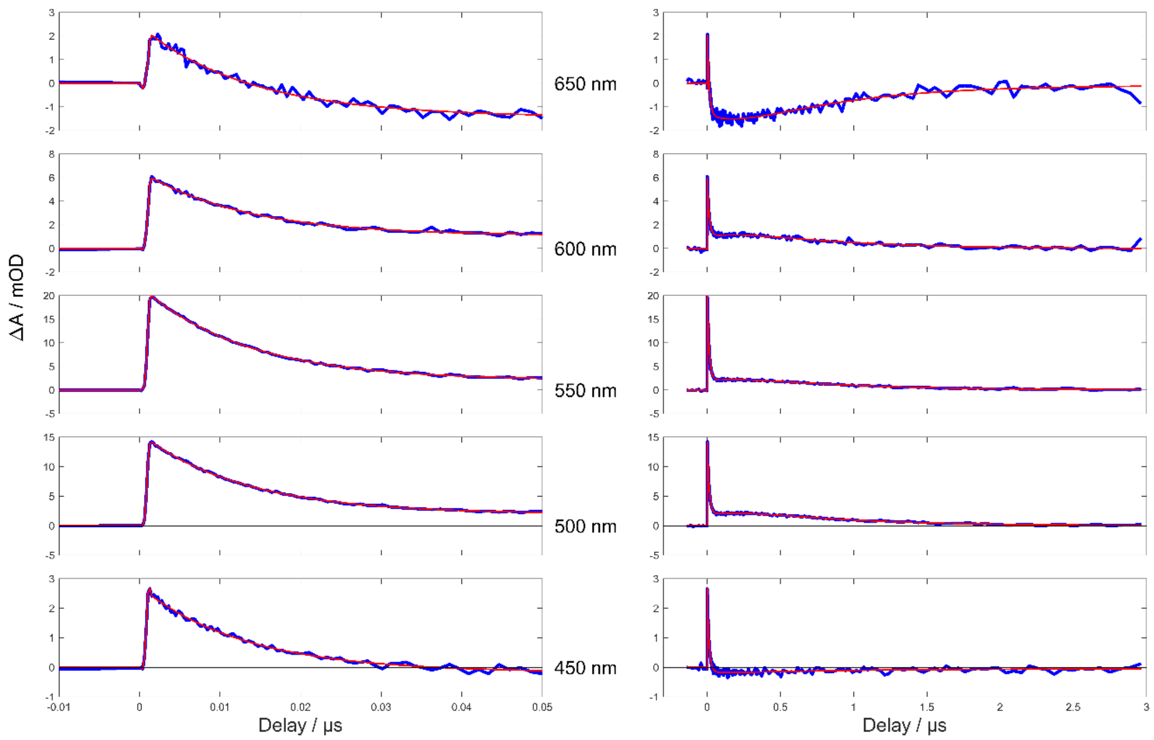

Supplementary Figure 13: *Selected kinetic traces (blue) with respective triexponential fits (red) for 0.5 mM $TDCl_4$ in toluene shown for initial 100 ns (left) and full timescale (3 µs).*

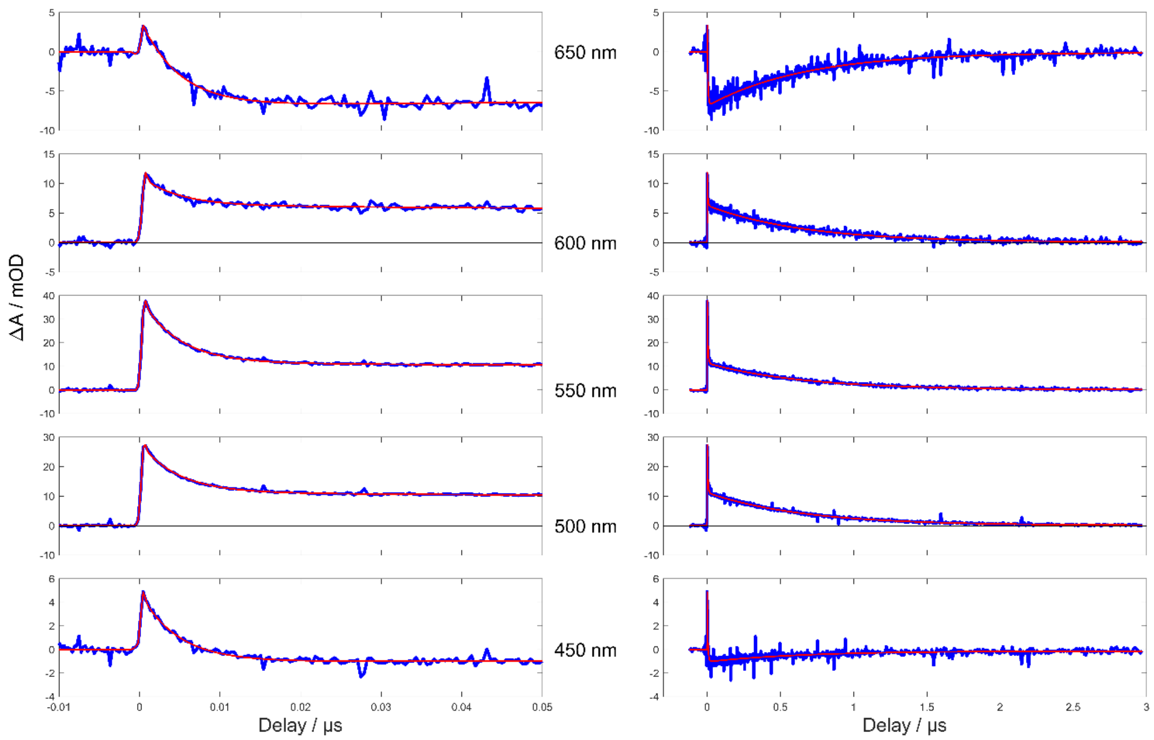

Supplementary Figure 14: *Selected kinetic traces (blue) with respective biexponential fits (red) for 40 mM $TDCl_4$ in toluene shown for initial 100 ns (left) and full timescale (3 µs).*

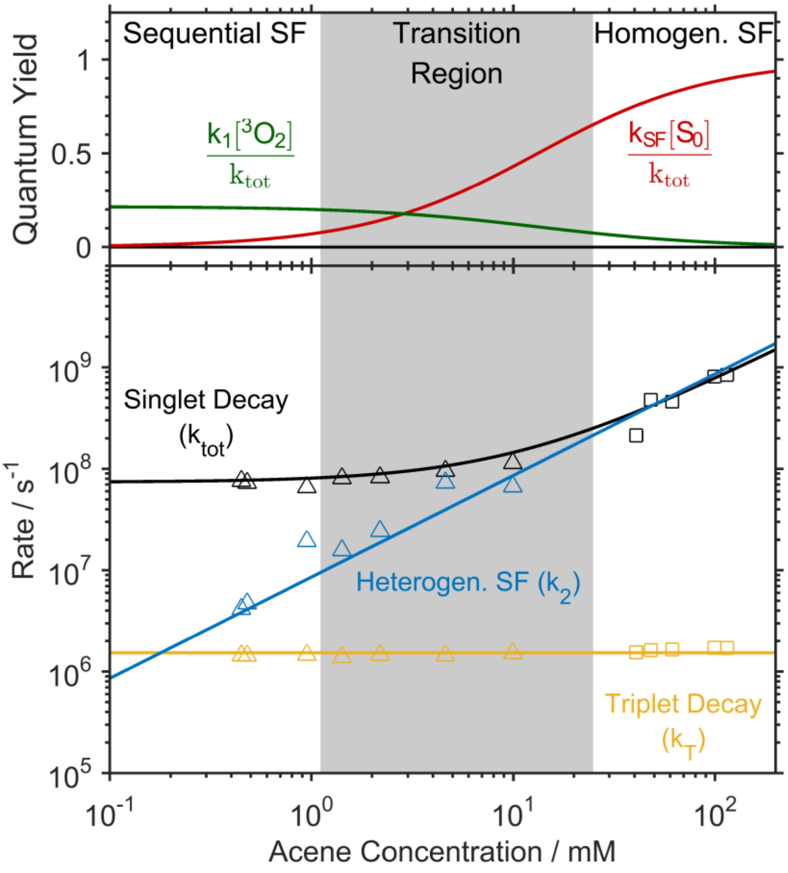

Supplementary Figure 15: *Rate constants for $TDCl_4$ obtained by a global multiexponential fit. For $c \leq 1$ mM, k_R has a concentration independent value of $(7.40\pm0.31) \times 10^7\ s^{-1}$, whereas for $c \geq 10$ mM, a linear fit yields a value of $(7.12\pm0.34) \times 10^9\ (Ms)^{-1}$, which is attributed to k_{SF}. The heterogeneous singlet fission constant amounts to $k_2 = (0.86\pm0.15) \times 10^{10}\ (Ms)^{-1}$ and is observed exclusively in a concentration range of 0.1 mM < c < 10mM. The triplet decays with a concentration independent rate of $k_T = (1.54\pm0.03) \times 10^6\ s^{-1}$.*

Species-Associated-Difference Spectra (SADS)

Inserting rate constants obtained by the global multiexponential fits for TIPS-Pn (Figure 5) and $TDCl_4$ (Supplementary Figure 15) into Eqns. 1 and 3 yields the pre-exponential coefficients needed to determine Singlet and Triplet SADS. Supplementary Figure 16 shows the respective results in concentration ranges for which k_2 was identified; normalized for singlet SADS intensity. In the case of TIPS-Pn (Supplementary Figure 16 a), the SADS show a good agreement over the whole concentration range. For $TDCl_4$, a slight deviation in the amplitudes of the triplet SADS is observed. However, it does not occur systematically depending on the concentration. Therefore, it can be attributed to the fact that singlet and triplet ESA have a strong spectral overlap, which hampers the disentanglement.

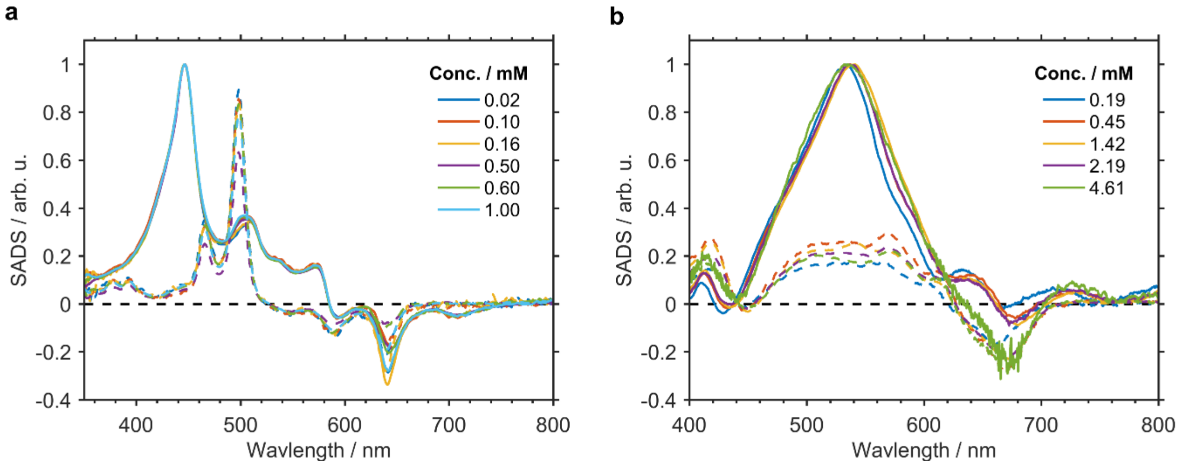

Supplementary Figure 16: *Singlet (solid lines) and triplet (dashed lines) SADS of a) TIPS-Pn and b)* $TDCl_4$ *for selected concentrations, obtained applying the mechanism proposed in Figure 2a and b. Over two orders of magnitude, only marginal differences are observable.*

Supplementary Note 6: Tabulated Rate Constants

Supplementary Table 1: *Results of the global multiexponential fits for TIPS-Pn shown in Supplementary Figure 9 – 11*

Concentration / mM	τ_1 / ns	τ_2 / ns	τ_3 / µs
**0.02**	11.5	-	2.02
**0.5**	12.3	242	1.88
**23**	7.40	-	2.39

Supplementary Table 2: *Results of the global multiexponential fits for $TDCl_4$ shown in Supplementary Figure 12- 14*

Concentration / mM	τ_1 / ns	τ_2 / ns	τ_3 / µs
**0.2**	14.4	260	1.04
**0.5**	13.6	214	0.70
**40**	4.88	-	0.65

Supplementary Table 3: *Rate constants of TIPS-Pn obtained by analysis of the multiexponential fit results over the concentration range of 0.02 – 146 mM:*

	Experimental Values	Literature
**$c(O_2)$**	-	1.81 mM[6]
**k_1**	$(1.04 \pm 0.13) \times 10^{10}$ $M^{-1}s^{-1}$	3.12×10^{10} $M^{-1}s^{-1}$ [7]
**k_2**	$(1.62 \pm 0.19) \times 10^{10}$ $M^{-1}s^{-1}$	3.12×10^{10} $M^{-1}s^{-1}$ [7]
**k_{SF}**	$(8.98 \pm 0.36) \times 10^{9}$ $M^{-1}s^{-1}$	2.18×10^{9} $M^{-1}s^{-1}$ [3]
**k_R**	$(8.57 \pm 0.19) \times 10^{7}$ s^{-1}	8.8×10^{7} s^{-1} [3]
**k_T**	$(6.55 \pm 0.30) \times 10^{5}$ s^{-1}	1.5×10^{5} s^{-1} [3]

Supplementary Table 4: *Rate constants of TDCl4 obtained by analysis of the multiexponential fit results over the concentration range of 0.2 – 115 mM:*

	Experimental Values	Literature
**$c(O_2)$**	-	1.99 mM[8]
**k_1**	$(1.03 \pm 0.16) \times 10^{10}$ $M^{-1}s^{-1}$	3.12×10^{10} $M^{-1}s^{-1}$ [7]
**k_2**	$(8.59 \pm 1.46) \times 10^{9}$ $M^{-1}s^{-1}$	3.12×10^{10} $M^{-1}s^{-1}$ [7]
**k_{SF}**	$(7.12 \pm 0.60) \times 10^{9}$ $M^{-1}s^{-1}$	-
**k_R**	$(7.40 \pm 0.31) \times 10^{7}$ s^{-1}	-
**k_T**	$(1.54 \pm 0.03) \times 10^{6}$ s^{-1}	-

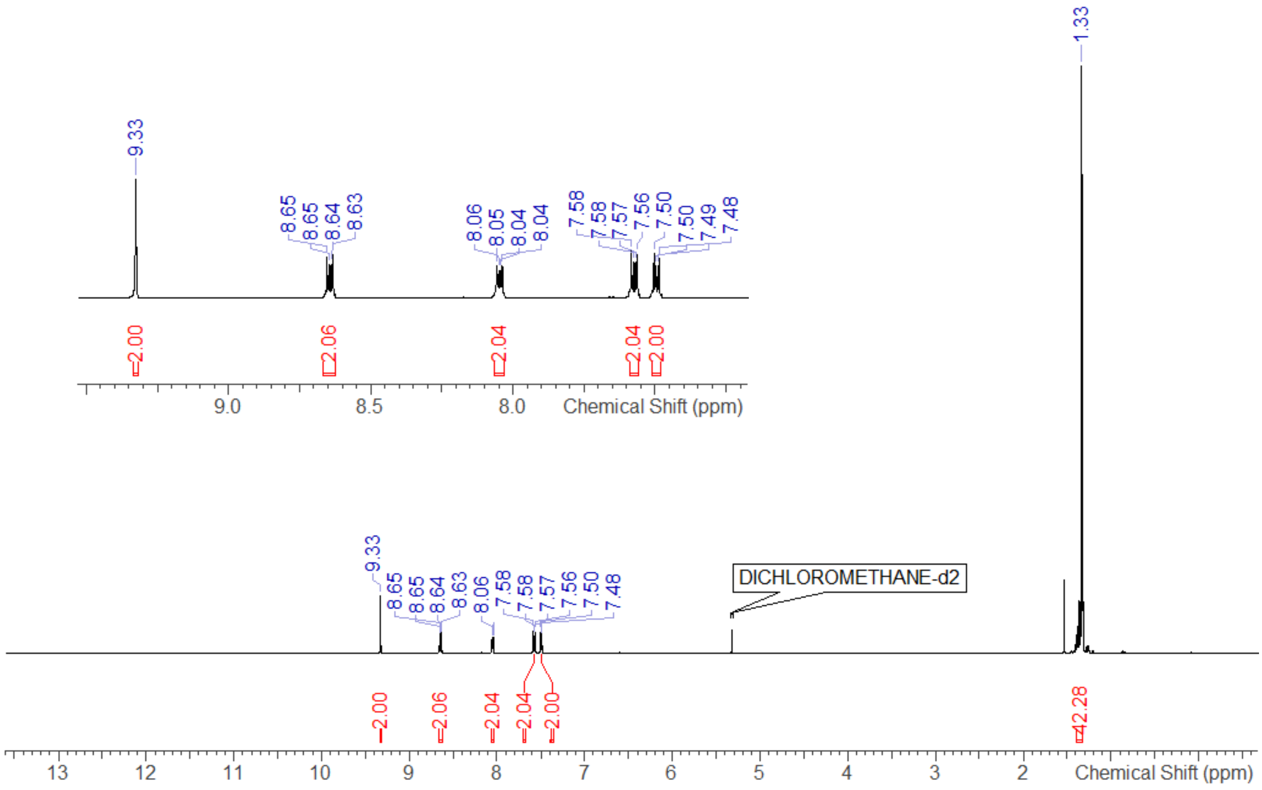

Supplementary Figure 17: *TIPS-Tetracene: 1H NMR (500 MHz, DICHLOROMETHANE-d2) δ 9.33 (s, 2H), 8.67 – 8.62 (m, 2H), 8.07 – 8.03 (m, 2H), 7.60 – 7.55 (m, 2H), 7.51 – 7.48 (m, 2H), 1.41 – 1.26 (m, 42H)*

Supplementary References

1. Einstein, A. Über die von der molekularkinetischen Theorie der Wärme geforderte Bewegung von in ruhenden Flüssigkeiten suspendierten Teilchen. *Annalen der Physik* **322**, 549-560, (1905).
2. Ware, W. R. Oxygen Quenching of Fluorescence in Solution: An Experimental Study of the Diffusion Process. *The Journal of Physical Chemistry* **66**, 455-458, (1962).
3. Walker, B. J., Musser, A. J., Beljonne, D. & Friend, R. H. Singlet exciton fission in solution. *Nature Chemistry* **5**, 1019-1024, (2013).
4. Aminabhavi, T. M. & Gopalakrishna, B. Density, Viscosity, Refractive Index, and Speed of Sound in Aqueous Mixtures of N,N-Dimethylformamide, Dimethyl Sulfoxide, N,N-Dimethylacetamide, Acetonitrile, Ethylene Glycol, Diethylene Glycol, 1,4-Dioxane, Tetrahydrofuran, 2-Methoxyethanol, and 2-Ethoxyethanol at 298.15 K. *Journal of Chemical & Engineering Data* **40**, 856-861, (1995).
5. Ogilby, P. R. & Foote, C. S. Chemistry of singlet oxygen. 42. Effect of solvent, solvent isotopic substitution, and temperature on the lifetime of singlet molecular oxygen (1.DELTA.g). *Journal of the American Chemical Society* **105**, 3423-3430, (1983).
6. Quaranta, M., Murkovic, M. & Klimant, I. A new method to measure oxygen solubility in organic solvents through optical oxygen sensing. *Analyst* **138**, 6243-6245, (2013).
7. Kearns, D. R. Physical and chemical properties of singlet molecular oxygen. *Chemical Reviews* **71**, 395-427, (1971).
8. Luehring, P. & Schumpe, A. Gas solubilities (hydrogen, helium, nitrogen, carbon monoxide, oxygen, argon, carbon dioxide) in organic liquids at 293.2 K. *Journal of Chemical & Engineering Data* **34**, 250-252, (1989).
